# Reduced perfusion in systemic sclerosis digital ulcers (both fingertip and extensor) can be increased by topical application of glyceryl trinitrate^☆^

**DOI:** 10.1016/j.mvr.2016.12.008

**Published:** 2017-05

**Authors:** M. Hughes, T. Moore, J. Manning, J. Wilkinson, G. Dinsdale, C. Roberts, A. Murray, A.L. Herrick

**Affiliations:** a Centre for Musculoskeletal Research, The University of Manchester, Salford Royal NHS Foundation Trust, Manchester Academic Health Science Centre, Manchester, United Kingdom; b Department of Rheumatology, Salford Royal NHS Foundation Trust, Salford, United Kingdom; c Research and Development, Salford Royal NHS Foundation Trust, Salford, United Kingdom; d Centre for Biostatistics, Institute of Population Health, School of Medicine, The University of Manchester, Manchester, United Kingdom; e Photon Science Institute, The University of Manchester, United Kingdom; f NIHR Manchester Musculoskeletal Biomedical Research Unit, Central Manchester NHS Foundation Trust, Manchester Academic Health Science Centre, United Kingdom

**Keywords:** Systemic sclerosis, Scleroderma, Digital ulcers, Digital ischaemia, Glyceryl trinitrate, Microvascular

## Abstract

**Objectives:**

In patients with systemic sclerosis (SSc), fingertip digital ulcers (DUs) are believed to be ischaemic, and extensor surface DUs a result of mechanical factors/microtrauma. Our aim was to assess blood flow response to topical glyceryl trinitrate (GTN) compared to placebo in SSc DUs, looking for differences in pathophysiology between fingertip and extensor lesions.

**Method:**

This was a double-blind, randomised, crossover, placebo-controlled study. Sixteen (6 fingertip, 10 extensor) DUs were each studied twice (one day apart): once with GTN and once with placebo ointment. Perfusion at the DU centre (‘DUCore’) and periphery (‘DUPeriphery’), as measured by laser Doppler imaging was performed before and immediately after ointment application, then every 10 min, up to 90 min post-application. We calculated the area under the response curve (AUC) and the ratio of peak perfusion to baseline, then compared these between GTN and placebo.

**Results:**

Perfusion was lower in the DUCore compared to the DUPeriphery (ratio of 0.52). The microvessels of the DUCore were responsive to GTN, with an increase in perfusion, with a similar effect in both fingertip and extensor DUs. The AUC and peak/baseline perfusion difference in means (ratio, 95% confidence interval) between GTN and placebo at the DUCore were 1.2 (1.0–1.6) and 1.2 (1.0–1.5) respectively, and at the DUPeriphery were 1.1 (0.8–1.6) and 1.0 (0.9–1.2) respectively.

**Conclusion:**

DUs (both fingertip and extensor) were responsive to topical GTN, with an increase in perfusion to the ischaemic DU centre. If both fingertip and extensor DUs have a (potentially reversible) ischaemic aetiology, this has important treatment implications.

## Highlights

• SSc fingertip DUs are believed to be ischaemic, whereas, extensor surface DUs are a result of mechanical factors/microtrauma.
• DUs (both fingertip and extensor) were responsive to topical GTN, in particular the ischaemic centre.
• If both fingertip and extensor DUs have a ischaemic aetiology, this has important treatment implications.

## 1 Introduction

Digital ulcers (DUs) are a major cause of pain and disability in patients with systemic sclerosis (SSc) (Bérezné et al., 2011, Mouthon et al., 2014), and are a biomarker of disease progression, including death (Mihai et al., 2016). However, relatively little is known about SSc-related DU pathophysiology. It is currently believed that fingertip DUs are ischaemia-driven, while those which occur over the extensor surfaces are related to mechanical abnormalities and microtrauma (Hachulla et al., 2007). Previous studies using laser-based techniques are supportive of an ischaemic component to DUs, with reduced perfusion to the centre of the DU compared to the periphery (Ruaro et al., 2015), including in extensor surface DUs (Murray et al., 2016). In addition, the tissue adjacent to DUs has been reported to be relatively hyperaemic compared to normal skin (Murray et al., 2016).

Whether DUs are ischaemic is a key clinical question because drug therapies, in general, rely upon vasodilation, to increase perfusion to the DU. A number of recent randomised controlled trials have excluded extensor DUs (Matucci-Cerinic et al., 2011, Hachulla et al., 2016, Khanna et al., 2016), presumably on the basis that if these ulcers are not ischaemic, then they are unlikely to benefit from vasoactive therapies.

Supplementation of the nitric oxide (NO) pathway is an important therapeutic strategy in the management of digital vascular disease in SSc (e.g. with phosphodiesterase type 5 inhibitors). In addition, we have previously reported that topical glyceryl trinitrate (GTN) (a NO donor) increases blood flow in *intact* SSc skin (Anderson et al., 2002) as measured by laser Doppler imaging (LDI).

Against this background, our aim was to assess the responsiveness of the microvessels in the centre (‘DU Core’) and adjacent tissue (‘DU Periphery’) of SSc-related DUs to topical NO donation from GTN, compared to placebo ointment, and whether this differs between fingertip and extensor DUs, to better inform our understanding of the pathogenesis (with therapeutic implications) of DUs in SSc. Our rationale was that if DU blood flow increases with GTN, then this would imply that the microvessels within DUs are capable of endothelial-independent vasodilation and that topical NO donation might be an effective therapy.

## 2 Patients and methods

### 2.1 Patients

Sixteen patients (13 female and 3 male) with SSc (7 lcSSc and 9 dcSSc) (LeRoy et al., 1988), with 6 fingertip and 10 extensor ulcers were studied. The mean (SD) age of patients was 55.7 (17.3) years. Raynaud's phenomenon (RP) duration (mean, SD) was 16.7 (8.8) years and disease duration (from first non-RP clinical manifestation) was 14.5 (8.6) years. All the patients had a history of previous DUs, often requiring previous prostanoid infusions (n = 11) and not uncommonly surgical debridement (n = 8) for severe digital vascular disease. SSc-associated autoantibodies were present in most patients, namely anticentromere (n = 3), anti-Scl-70 (n = 7) and anti-RNA polymerase III (n = 2). The majority of patients were receiving current vasodilatory treatment/s (n = 14), most commonly with calcium-channel blockers (n = 10), and with several patients receiving treatment with an endothelial receptor antagonist (n = 3) or PDE5-inhibitior (n = 1). The study was approved by the National Research Ethics Committee – Preston and all patients provided signed informed consent.

We did not use a particular DU definition in the study. Previous clinical trials have used different DU definitions: we adopted a pragmatic, ‘real-world’ approach. Two clinicians (MH and AH) with an interest in SSc-related DUs assessed the lesions prior to recruitment into the study. Salford Royal NHS Foundation Trust is a tertiary referral centre for SSc (including participation in previous DU clinical trials), and many of the patients included in the study had a history of severe digital vascular disease. Therefore, taken together, it is likely that the patients in our study were representative of those who would be included in SSc-related clinical trials. Patients with eschar overlying the DU were included, if the clinician felt the thickness was only minimal, and unlikely to have any detrimental effect on the potential impact of the ointments.

### 2.2 Study protocol

Patients were randomised (with a balanced allocation within DU subgroup - fingertip versus extensor) to receive either GTN or placebo ointment on day one, and the alternative the following day. Sealed envelopes contained the patients' allocation schedule, allowing the medication to be dispensed. Patients, and the two operators, one of whom applied the study medication and the second of whom performed the LDI (and later extracted LDI data), were all blinded to the randomisation. This approach with two operators minimised any bias from any potential information gained from applying the ointment (e.g. patient opinion, including any side effects). One patient received intravenous iloprost on day one after the first study visit; however, the second visit was over 12 h after completion of the infusion.

Patients were advised to refrain from caffeine containing beverages and smoking for a period of at least 4 h before each study visit. No changes were made to patients' existing vasodilatory therapy. After a 20 minute period of acclimatisation at 23 °C, baseline LDI of the DU was performed, using a modified MoorLDI-vr (Moor Instruments, Axminster, United Kingdom) LDI (red, 633 nm). Immediately after the initial image (or ‘flux map’) was acquired, either 200 mg GTN (2% Percutol ®) or placebo ointment (of similar appearance and consistency to GTN preparation) was applied to the DU, using a sterile applicator, and with a circular motion, for 1 min. Any visible excess ointment was promptly removed using gauze. LDI was performed immediately (time 0) after application of the ointment, and then every 10 min, up to 90 min, at each study visit. Imaging was terminated if the patient indicated a desire to stop. Patients were asked to report any side effects experienced during the study visits.

### 2.3 Image analysis

**Fig. 1:**
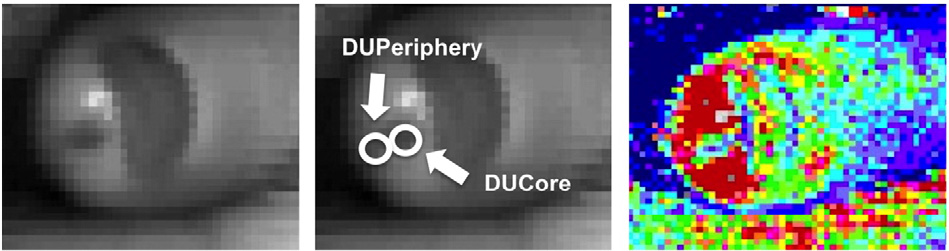
LDI regions of interest. Illustration of how the regions of interest (ROI) were extracted to measure DU perfusion. Left and middle: identical grey scale images of a fingertip DU, the middle image illustrate the ROI of the DUCore and DUPeriphery. Right: The corresponding LDI perfusion (flux map) image of the DU. Blue indicates low perfusion, whereas, red is relatively higher perfusion. The perfusion to the DUCore is lower (i.e. ischaemic) compared to the DUPeriphery.

Perfusion data were extracted from the captured images. Using the LDI grey-scale image of the DU (to avoid bias from seeing the perfusion image), regions of interest (Fig. 1) of the same size were created to extract perfusion data from the DUCore and the DUPeriphery for each treatment visit. We chose to examine the DU adjacent skin because of the relative hyperaemia (as previously described), which could be important in DU healing.

### 2.4 Statistical analysis

Summary measures of each patient's response to GTN and placebo: area under the curve (AUC) and ratio of peak perfusion compared to baseline were calculated. We calculated the difference in means and a 95% confidence interval (95% CI) for each of these summary measures, accounting for the correlation between paired measurements. For both measures we calculated the difference in means and 95% CI on a logarithmic scale due to distributional skewness, before back-transforming to the original scale. This yielded values representing the ratio of GTN response compared to placebo. For two patients who did not complete the full observation period, we calculated AUC and peak/baseline perfusion restricted to the same amount of time in the comparator period, to ensure a like-for-like comparison. All statistical analyses were performed using STATA version 13.

## 3 Results

**Fig. 2:**
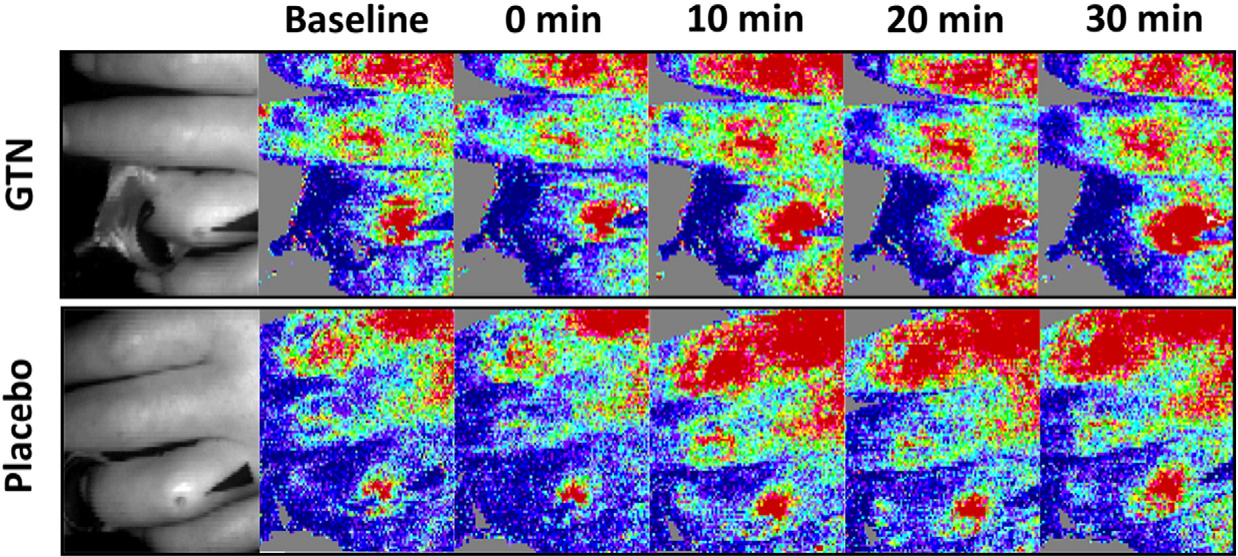
DU LDI. Example of LDI perfusion data for an extensor DU with placebo (top panel) and glyceryl trinitrate (GTN) (bottom panel). Grey scale images of the DU on the respective days are provided on the left hand section of the panels. LDI perfusion data is presented prior (baseline), immediately after (0 min) application of the study ointment, and then every 10 min for 30 min. GTN compared to placebo was associated with a marked increase in perfusion to the DUCore and DUPeriphery.

Fourteen (6 fingertip and 8 extensor) DUs were included in the final analysis. LDI perfusion data could not be extracted for two extensor DUs, or from the GTN day for a third extensor DU, because the DU could not be confidently identified on the grey-scale images. An example of the LDI perfusion data (flux maps) for an extensor DU at baseline and up to 30 min post application of GTN and placebo is provided in Fig. 2. In most (n = 8) DUs all the planned LDI measurements were completed on both days. In three patients a single LDI measurement (i.e. at one time point only e.g. 60 min) was unavailable for analysis on either one or both of the days. Three patients had incomplete LDI data (i.e. fewer measurements than intended in the study design) and/or LDI was performed at different (but known and recorded) time points; however, these data were still included in the final analysis. The reasons for these deviations in study protocol were either due to technical issues (e.g. LDI equipment failure) and/or patient preference.

Two patients reported local side effects (e.g. pain and dysesthesia) after application of GTN ointment: these were mild, and did not require any action. One patient experienced marked local and systemic vasodilatory side effects (including a sensation of light headedness), with an objective (by LDI) increase in perfusion of the other digits, although this did not necessitate discontinuation of the study.

### 3.1 DU response to GTN and placebo

**Fig. 3:**
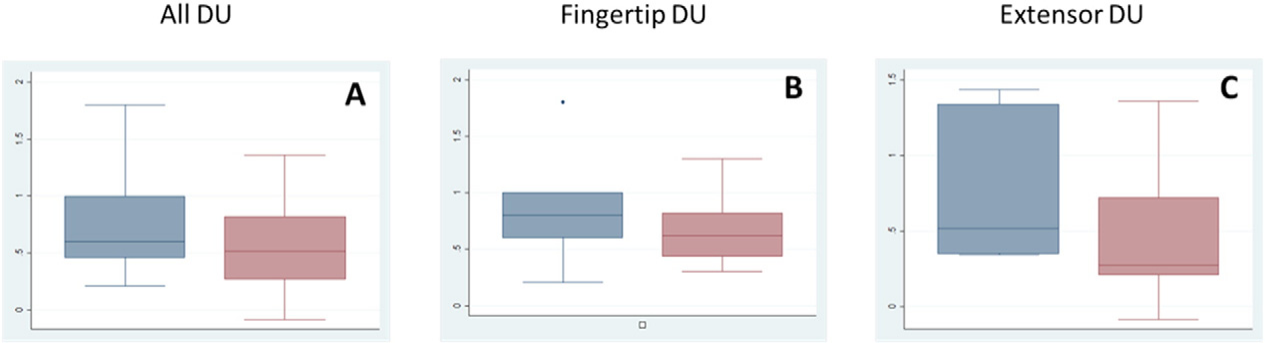
Peak to baseline perfusion: GTN vs placebo. Box plots are provided for the peak/baseline perfusion (log transformed) for the DUCore. The responses of all (A), fingertip (B) and extensor (C) DUs to GTN (blue) and placebo (pink) are presented. Median and interquartile ranges are displayed (boxes) together with the range (whiskers).

**Fig. 4:**
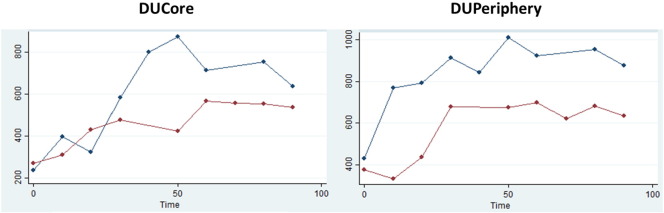
DU perfusion curve. An example of characteristic perfusion curves for both the DUCore and DUPeriphery, demonstrating a marked increase in perfusion with both GTN (blue) and placebo (red). Perfusion (left hand axis) measured in arbitrary perfusion units.

At baseline, all 14 DUs had reduced perfusion in the core relative to the periphery, with a mean (standard deviation) ratio of DUCore/DUPeriphery = 0.52 (0.27), including both fingertip (0.44 [0.29]) and extensor (0.59 [0.26]) DUs. Table 1 presents the summary measures broken down by DU type and treatment. Fig. 3 depicts box plots describing the peak/baseline perfusion ratios for the DUCore. Median (IQR) time to maximum perfusion for GTN and placebo at the DUCore was 50 (30 to 60) min and 30 (10 to 50 min) respectively, and at the DUPeriphery 50 (10 to 70) min and 40 (20 to 70) min respectively. An example of characteristic perfusion curves is provided in Fig. 4.

## 4 Discussion

The key finding of this study is that we have demonstrated the responsiveness of the DU microvessels to GTN compared to placebo at the centre of the DU, and that this response occurs in both fingertip and extensor DUs. In addition, all the analysed DUs had relatively low perfusion at their centres when compared to the periphery, further confirming that DUs are likely to be ‘ischaemic’ in aetiology. These findings have important clinical implications, namely: if extensor (similarly to fingertip) DUs have a *potentially reversible* ischaemic aetiology, they might benefit from treatment with vasoactive therapies (including local NO donors) to increase blood flow and likely improve DU healing, in the same way as fingertip ulcers. This could warrant the inclusion of extensor DUs into future clinical trials.

Our data further support that the tissue adjacent to the DU is relatively hyperaemic, the pathophysiology and significance of which remains unclear. There was heterogeneity in the response by DUs (as evidenced by relatively large standard deviations relative to the mean), which could suggest that a spectrum of relative DU ischaemia might exist. In SSc, although impaired endothelial-dependent vasodilation has been fairly consistently reported, some studies have found that endothelial-independent vasodilation is also compromised (Anderson et al., 2004, Gunawardena et al., 2007). Therefore, in some patients, DUs might be unable respond to NO supplementation, due to failure of endothelial-independent vasodilation, which has important therapeutic implications.

Topical GTN has previously been investigated as a ‘local’ treatment for RP (including SSc-related RP) and is currently being revisited (Hummers et al., 2013). In a small crossover study of topical GTN for secondary RP (mainly SSc), Franks (1982) observed there was a marked improvement in DUs with GTN, but not with placebo. In our study we did not expect to observe any potential ‘therapeutic’ (i.e. healing) effect with a single dose of GTN. However, the increased DU perfusion from GTN is encouraging, and suggests that supplementation of the NO pathway could be a promising target to be re-explored in future research. However, of note, GTN is commonly associated with systemic effects, as observed in one patient in this study, and so the dose must be carefully selected: sufficient to cause local vasodilation, but not large enough to result in systemic absorption and the risk of vasodilatory side effects. Another important point is that current preparations of topical GTN are fairly unpleasant (e.g. greasy) for patients to apply, and so new, ‘user-friendly’ formulations are required.

An increase in perfusion at both the DU centre and periphery was observed with placebo ointment. In our previous study (Anderson et al., 2002), application of placebo ointment was associated with an increase in skin perfusion compared to an untreated finger, although this was less marked than with GTN and also shorter lived. This is most likely caused by the direct action of massaging the placebo ointment onto the skin. Furthermore, the peak to perfusion was reached earlier with placebo compared to GTN (median time 30 and 50 min, respectively). In future proof of concept studies of topical treatments for SSc-related DUs, it will be important to consider the impact of the increased perfusion from application of placebo alone, to enable any ‘active’ treatment effect to be established.

Our study has a number of important considerations. Although this was a small study, it benefited from a robust experimental design (double-blind, randomised, crossover, placebo-controlled), with objective physiological measurements. We used a single dose of 2% GTN to study DU pathophysiology, as in our previous study (Anderson et al., 2002) in which a similar dose of GTN increased perfusion in intact SSc skin. However, in future studies, different dose/s of GTN could be considered. In addition, it will be important to examine the degree of *within* patient variation to GTN as our data suggests that DU response is heterogeneous, and to determine whether a ‘ceiling’ effect exists, where the microvessels display no additional response to GTN. Furthermore, as previously described, future studies will need to be of long enough duration to capture the peak in perfusion from GTN compared to placebo (the placebo peak occurred earlier in our study).

In conclusion, the microvessels of both fingertip and extensor DUs were responsive to GTN compared to placebo, particularly in the relatively ischaemic DU centre. Further research is warranted to understand the ischaemic drive to the formation of *all* DUs, including extensor aspect DUs, and whether this is potentially *reversible* (including through topical NO donation). This has important treatment implications, including on the design of future clinical trials.

## Funding

Arthritis Research UK (grant references 20482 and 19465). This report includes independent research supported by the National Institute for Health Research Biomedical Research Unit Funding Scheme. The views expressed in this publication are those of the author(s) and not necessarily those of the NHS, the National Institute for Health Research or the Department of Health.

## Figures and Tables

**Table 1:** Summary statistics for the DUCore and DUPeriphery. Data are presented as mean (standard deviation). The area under curve (AUC) and ratio of peak perfusion compared to baseline are presented for all, fingertip and extensor DUs. Comparisons are the ratio of means (95% CIs) for the AUCs and peak perfusion/baseline ratios (GTN:placebo). Ratios with values greater than 1 indicate an increased response from glyceryl trinitrate (GTN) compared to placebo (PBO).

	All digital ulcers			Fingertip digital ulcers			Extensor digital ulcers		
	GTN	PBO	Comparison	GTN	PBO	Comparison	GTN	PBO	Comparison
DUCore									
AUC (arbitrary perfusion units)	26,245.42 (19,829.5)	23,973.97 (18,607.44)	1.2 (1.0 to 1.6)	18,453.6 (10,721.8)	16,506.8 (12,498.3)	1.3 (0.7 to 2.2)	32,924.1 (24,038.9)	29,574.3 (21,161.2)	1.2 (1.0 to 1.6)
Peak perfusion/baseline perfusion (ratio)	2.5 (1.4)	1.9 (0.9)	1.2 (1.0 to 1.5)	2.7 (1.7)	2.1 (0.8)	1.2 (0.8 to 1.9)	2.3 (1.2)	1.8 (1.0)	1.2 (1.0 to 1.6)

DUPeriphery									
AUC (arbitrary perfusion units)	62,025.2 (41,910.7)	52,185.4 (33,734.3)	1.1 (0.8 to 1.6)	51,939.1 (42,923.2)	49,019.0 (32,584.1)	0.9 (0.4 to 2.0)	70,670.4 (42,293.6)	54,560.3 (36,606.2)	1.4 (1.1 to 1.7)
Peak perfusion/baseline perfusion (ratio)	2.2 (1.5)	2.1 (1.4)	1.0 (0.9 to 1.2)	2.4 (2.2)	2.5 (2.1)	0.9 (0.7 to 1.2)	2.0 (0.7)	1.8 (0.8)	1.1 (1.0 to 1.4)
